# Risk factors of postoperative pulmonary complications after primary posterior fusion and hemivertebra resection in congenital scoliosis patients younger than 10 years old: a retrospective study

**DOI:** 10.1186/s12891-022-05033-1

**Published:** 2022-01-26

**Authors:** Lulu Ma, Xuerong Yu, Jianguo Zhang, Jianxiong Shen, Yu Zhao, Shugang Li, Yuguang Huang

**Affiliations:** 1 Department of Anesthesiology, Peking Union Medical College Hospital, No 1, Shuaifu Yuan, Dongcheng District, Beijing, 100730 China; 2 Department of Orthopedics, Peking Union Medical College Hospital, No 1, Shuaifu Yuan, Dongcheng District, Beijing, 100730 China

**Keywords:** Congenital scoliosis, Osteotomy, Postoperative pulmonary complication, Thoracoplasty

## Abstract

**Background:**

Postoperative pulmonary complications are common and associated with morbidity and mortality. Congenital scoliosis is a failure of vertebral formation and/or segmentation arising from abnormal vertebral development. Posterior fusion and osteotomy are necessary for these patients to prevent deterioration of spine deformity. The incidence of postoperative pulmonary complications in this specific group of patients, especially young children were unknown.

**Methods:**

A retrospective study was conducted and electronic medical records of early-onset scoliosis patients who had primary posterior fusion and hemivertebra resection at our institution from January 2014 to September 2019 were reviewed. The demographic characteristics, the intraoperative and postoperative parameters were collected to identify the predictors of postoperative pulmonary complications.

**Results:**

A total of 174 patients (57.5% boys) with a median age of 3 years old were included for analysis. Eighteen patients (10.3%) developed perioperative pulmonary complications and pneumonia (*n*=13) was the most common. History of recent upper respiratory infection was not related to postoperative pulmonary complications. Multifactorial regression analysis showed thoracoplasty was the only predictive risk factor of postoperative pulmonary complications.

**Conclusions:**

For congenital scoliosis patients younger than 10 years old, thoracoplasty determine the occurrence of postoperative pulmonary complications. Both surgeons and anesthesiologists should pay attention to patients undergoing thoracoplasty and preventive measures are necessary.

## Background

Congenital scoliosis is a failure of vertebral formation and / or segmentation arising from abnormal vertebral development. If left untreated, the severity of spine deformity may progress, and cardiopulmonary function and the quality of life will be impaired. Posterior spinal fusion w/o osteotomy are necessary for these patients to prevent the deterioration of vertebral severity.

Postoperative pulmonary complications (PPC) are common and associated with morbidity, mortality, Intensive Care Unit (ICU) admission and higher costs [1, 2]. The incidence of PPC ranged from 2.0 to 5.6% [3, 4]. Pulmonary complications are not uncommon after scoliosis surgery [5]. Pulmonary and functional restriction start early in mild idiopathic scoliosis [6]. And the progression of scoliosis is responsible for the deformity of rib cage and deterioration of pulmonary function. Pulmonary function will further deteriorate in the immediate postoperative period [7]. Previous reports had demonstrated that large Cobb angles [8, 9], long operation times [8], blood transfusion [8], thoracoplasty [10] and decreased forced vital capacity and forced expiratory volume in one second [9] were risks factors for PPC after scoliosis surgery. Age has also been confirmed as the risk factor for respiratory critical events, with 12% decrease for each increasing year of age [11].

There are few literatures about the incidence and risk factors of pulmonary complications in congenital scoliosis patients especially in patients younger than 10 years old. The aim of this study was to determine the incidence and risk factors of PPC after primary posterior fusion and hemivertebra resection in patients younger than 10 years old.

## Methods

Our study was approved by institutional review board of Peking Union Medical College Hospital and the need for informed consent was waived by institutional review board of Peking Union Medical College Hospital given its retrospective design. Electronic medical records of patients who underwent primary posterior fusion and hemivertebra resection at our institution from January 2014 to September 2019 were reviewed. The exclusion criteria included: diagnosis inconsistent with congenital scoliosis and hemivertebra, patients who had vertebral resection, age older than 10 years, revision surgery, and those with incomplete data.

The indication of hemivertebra resection in our institution were as followings: (1) curve magnitude >25°with fast progression, (2) progression of curve >5°in 6-month follow-up, (3) failure of conservative treatment. Resection was performed by four surgeons at the department of Orthopedics, Peking Union Medical College Hospital. All patients had the operation under general anesthesia. Continuous infusion of propofol and remifentanil was used to maintain general anesthesia and fentanyl bolus (1-2ug/kg) was given for intraoperative analgesia. Transcranial motor evoked potentials were monitored intraoperatively. The number of fused levels was determined by the position of hemivertebra and the severity of scoliosis or kyphosis. Details of surgical technique had been described in our previous work [12]. Thoracoplasty was indicated in patients who had significant thoracic deformity with a rib hump height difference of >3cm or a hump angle of >10°.

PPC were defined as the development of atelectasis, pleural effusion, pneumothorax, pneumonia, hypoxemia (SpO2< 90% or PaO2<60mmHg in room air) and increased requirement for postoperative ventilatory support. Postoperative chest radiographs or thoracic ultrasound were performed to detect the occurrence of atelectasis, pneumonia, pleural effusion or pneumothorax in patients with abnormal symptoms or signs or abnormal chest auscultation. Drainage was placed depending on patients’ symptoms and the degree of effusion or pneumothorax. And antibiotics were administrated for the treatment of pneumonia after assessment by pediatrician.

The following data were collected, which included age, sex, weight, comorbidities, other congenital deformities, history of recent upper respiratory infection (within two weeks before operation), duration of anesthesia and operation, perioperative transfusion, admission to Intensive Care Unit (ICU), the length of hospital stay and postoperative pulmonary complications. Scoliosis was measured using Cobb method and the details of measurement had been described in our previous article [12]. The major curve was used for analysis in patients with more than one curve.

Data were analyzed using SPSS 24.0(IBM Corporation). Continuous variables were presented as means and standard deviations or medians and interquartile ranges. Category variables were presented as numbers and percentages. Patients were divided into two groups depending on whether they had PPC. Student t test or Wilcoxon rank sum test was used to compare variables between two groups. Binary logistic regression analysis was used to determine predictive risk factors of PPC.

## Results

A total of 174 patients were included for analysis. There were 100 boys and 74 girls. The median age was 3 [2, 5] years old. 18 patients had congenital heart disease, 9 patients had congenital digestive deformities, 6 patients had congenital urological deformities and 6 patients had congenital neural deformities. Fourteen patients had rib anomalies, which included 11 cases of fused ribs, 4 cases of defect of ribs, and 2 cases of forked ribs variations.

The mean segmental cobb angle, total Cobb angle and kyphosis angle were 40+12°, 35+13° and 20+16 ° respectively. The median fused levels were 4. One hundred fifty-three patients had one hemivertebra resected, while 21 patients had 2 hemivertebra resected.

**Fig. 1:**
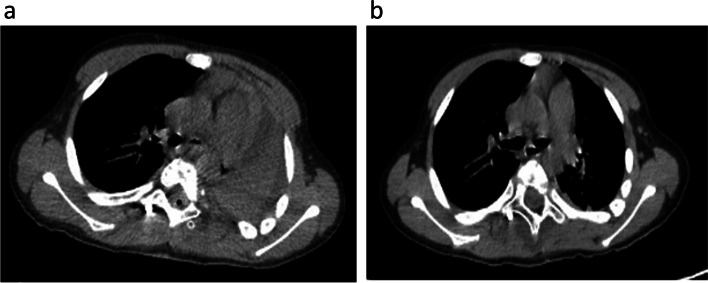
A 9-year-old male boy developed complete left atelasis and bronchoscopy was needed for lung expansion. He had C7 and T2/3 hemivertebra resection and posterior fusion. He developed tachypena and tachycardia on postoperative day 1. Physical examination showed asymmetric thoraci movement, absent left breath sound. And chest CT showed complete left pulmonary atelasis and pleura effusion (See Figure **a**). He had brochoscopy under general anesthesia and bronchoscopy revealed sputum completely occluded left main bronchus. Figure **b** showd left lung expanded after the sputum plug was removed

There were 25 documented PPC in 18 patients and the incidence of postoperative pulmonary complications was 10.3%. Pneumonia (*n*=13) was the most common postoperative complication, followed by pleural effusion (*n*=5), atelectasis (*n*=4), and increased requirement for postoperative ventilatory support (*n*=3). Two patients need drainage for pleural effusions and one need bronchoscopy. One patient was admitted to ICU due to desaturation after extubation for prolonged ventilatory support. Figure 1 showed a patient who developed complete left atelasis and bronchoscopy was needed for lung expansion.

Patients were divided into two groups depending on the occurrence of PPC. There was no difference in sex distribution, age, weight, other congenital deformities, rib anomalies, segmental Cobb angle, total Cobb angle, correction of segmental, total Cobb angle and kyphosis (See Table 1).

**Table 1:** Characteristics of patients with or without postoperative pulmonary complications

	Total (*n*=174)	Post pulmonary complications		*p*
		Yes (*n*=18)	No (*n*=156)	
Sex				
Male	100 (57.5%)	11 (61.1%)	89 (57.1%)	0.806
Female	74 (42.5%)	7 (38.9%)	67 (42.9%)	
Age ( years old)	3 [2,5]	4.5 [2.75,6.25]	3 [2,5]	0.342
Weight (Kilogram)	18.0+6.5	19.9+8.1	17.8+6.3	0.199
History of recent upper respiratory infection	22 (12.6%)	3 (16.7%)	19 (12.2%)	0.705
Other congenital deformities				
Congenital heart disease	18(10.3%)	2(11.1%)	16 (10.3%)	0.806
Congenital digestive deformities	9 (5.2%)	0	9 (5.8%)	0.600
Congenital urological deformities	6 (3.4%)	1 (5.6%)	5 (3.2%)	0.486
Congenital neural deformities	6 (3.4%)	0	6 (3.8%)	1.000
Other	7 (4.0%)	0	7 (4.5%)	1.000
Rib anomalies				0.595
Fused ribs	11 (6.3%)	2 (11.1%)	9 (5.8%)	
Defect of ribs	3 (1.7%)	0	3 (1.9%)	
Forked ribs	2 (1.1%)	0	2 (1.3%)	
Segmental Cobb angle (°)	40+12	44+14	40+12	0.184
Total Cobb angle (°)	35+13	39+13	34+12	0.162
Kyphosis (°)	20+16	25+19	19+16	0.170
Correction of totalCobb angle (%)	76.5+19.3	74.2+19.8	76.7+19.2	0.590
Correction of segmental Cobb angle (%)	73.3+22.3	74.9+26.4	73.1+21.8	0.743
Correction of kyphosis (%)	57.2+48.8	74.9+59.7	55.2+47.2	0.104
Main curve				0.120
Thoracic	37 (21.3%)	7 ((38.6%)	30 (19.2%)	
Thoracolumbar	59 (33.9%)	6 (33.3%)	53 (34.0%)	
Lumbar	78 (44.8%)	5 (27.8%)	73 (46.8%)	

The location of main curve was similar in both groups (*P*=0.120) and patients who developed PPC were more likely to have cervical, upper and middle thoracic screws (See Table 2). Patients who had PPC had longer anesthesia duration (*P*=0.001), longer operation time (*P*=0.030), and longer hospital stay (*P*<0.001). Intraoperative blood loss (*P*=0.003), transfusion (*P*<0.001) and the proportion of thoracoplasty (*P*<0.001) were higher in patients with PPC.

**Table 2:** Intraoperative and postoperative data in patients with or without complications

	Total (*n* = 174)	Pulmonary complications		*p*
		Yes (*n* = 18)	No (*n* = 156)	
No of levels fused	4 [2, 5]	5 [3.75, 6.25]	4 [2, 5]	0.015
No of hemivertebra resected				0.240
One	153 (87.9%)	14 (77.8%)	139 (89.1%)	
Two	21 (12.1%)	4 (22.2%)	17 (10.9%)	
Cervical screw	4 (2.3%)	3 (16.7%)	1 (0.6%)	0.004
Upper thoracic screw	16 (9.2%)	5 (27.8%)	11 (7.1%)	0.014
Middle thoracic screw	40 (23.0%)	8 (44.4%)	32 (20.5%)	0.035
Lower thoracic screw	88 (50.6%)	9 (50%)	79 (50.6%)	1.000
Lumbar screw	129 (74.1%)	10 (55.6%)	119 (76.3%)	0.084
thoracoplasty	11 (6.3%)	6 (33.3%)	5 (3.2%)	<0.001
Duration of anesthesia (minute)	229+45	262+65	225+41	0.001
Duration of operation (minute)	180+40	200+45	178+39	0.030
Intraoperative blood loss (ml)	268+233	423+531	250+162	0.003
Transfusion	113 (64.9%)	14 (77.8%)	99 (63.5%)	<0.001
Length of stay (day)	7 [6,8]	9.5 [7,13]	7 [6,8]	<0.001

Binary Logistical regression showed thoracoplasty (Odds Ratios 8.985, 95% Confidence Interval 1.019-79.259, *p*=0.048) was the only independent risk factors of perioperative pulmonary complications in patients younger than 10 years old undergoing primary posterior fusion and hemivertebra resection.

## Discussion

In this retrospective study of patients younger than 10 years old undergoing primary hemivertebra resection and fusion, we did find thoracoplasty was the independent risk factor of PPC. And patients with postoperative pulmonary complications also had prolonged length of stay in hospital.

Respiratory complications are the most common complications in pediatric patients [11] and are associated with poor outcome and higher costs [13]. Factors related to patients, anesthesia, and surgery all contribute to perioperative respiratory adverse events [14]. Younger age, pulmonary comorbidity, preoperative pulmonary function, endotracheal intubation, intravenous anesthesia, experience of anesthesiologist have been confirmed to be associated with increased risk of respiratory complications [15–17]. The incidence of PPC was 10.3% in our study, which was similar to previous reports [18, 19].

Thoracoplasty was the independent risk factor of perioperative pulmonary complications in our study. Thoracoplasty is the treatment of rib hump, which includes resection of several ribs and reduce the severity of a rib hump. And it has been proven as the predictive risk factors of perioperative pulmonary complications after posterior instrumentation and fusion for non-degenerative scoliosis [18, 20], adolescent idiopathic scoliosis [21] and patients with compromised pulmonary function [10]. Thoracoplasty was associated with significant decrease of immediate [22] and long-term postoperative pulmonary function [23]. However, in patients with neuromuscular scoliosis [24], thoracoplasty was not correlated with the worse outcome. All these findings suggest the necessity of weighing benefits and risks before performing thoracoplasty in scoliosis of different etiologies.

Recent upper respiratory infection (URI) is still a common reason for the postponement of pediatric elective surgery. The incidence of airway adverse events is high in pediatric patients with an active URIs [15, 25], however it is now general accepted that it is no longer necessary to postpone surgery for a period of 6 weeks [14]. And it is safe for patients with URIs undergoing procedural sedation [26] or general anesthesia. Although we did not find the relationship between the history of upper respiratory infection and pulmonary complications, the decision to continue the operation without postponement should be made carefully after weighing the benefits and risks and experienced anesthesiologists are necessary.

Pulmonary function test is widely used for preoperative pulmonary evaluation. However it was not routinely tested in our series of patients, since the median age was 4 years old and they were unlikely to cooperate the examination of pulmonary function test. Its role in the preoperative evaluation of scoliosis patients is controversary. No association had not been found to be associated with pulmonary function and postoperative intubation or intensive care unit admission in scoliosis children undergoing posterior spinal fusion [27]. But preoperative pulmonary function was the sensitive predictor of PPC in patients with neuromuscular scoliosis [9]. The role of pulmonary function in pediatric patients of congenital scoliosis needs further research.

There were several limitations of this study. First this is a retrospective study conducting at a single center. Variations in surgical skills, anesthetic techniques and criteria of extubation or admission to ICU may have influences on our results. Second, the number of patients who developed PPC was small and this might limit our ability to detected statistically significant factors. Third, chest radiographs or ultrasound were only obtained in patients with abnormal cardiopulmonary symptoms or chest auscultation findings and the prevalence of PPC might be underestimated.

In conclusion, for congenital scoliosis patients who were younger than 10 years old, thoracoplasty is the only predictor for perioperative pulmonary complications after posterior fusion and hemivertebra resection. Surgeons should balance the benefits and risks when making the decision of thoracoplasty.

## Data Availability

Raw data would be made available on reasonable request and with the permission of the institution where the data were generated. Xuerong Yu was the person to be contacted if someone wants to request the data from this study.

## Abbreviations

PPC: Postoperative pulmonary complications
ICU: Intensive Care Unit
RUI: Recent upper respiratory infection
